# TrainTracks - federated learning for reproducible research on sensitive medical data

**DOI:** 10.1186/s12911-026-03553-7

**Published:** 2026-05-21

**Authors:** Mayra Elwes, Mehrshad Jaberansary, Fu-Sung Kim-Benjamin Tang, Markus Aswendt, Oya Beyan, Ekaterina Kutafina

**Affiliations:** 1 Institute for Biomedical Informatics, Faculty of Medicine, University Hospital Cologne, Kerpener str. 62, 50937 Cologne, North-Rhine-Westphalia Germany; 2 Department of Neurology, Experimental Neurology Section, Goethe University Frankfurt and University Hospital, Theodor-Stern-Kai 7, 60596 Frankfurt am Main, Germany; 3 Department for Neurology, Faculty of Medicine, University Hospital Cologne, Kerpener str. 62, 50937 Cologne, North-Rhine-Westphalia Germany; 4 Fraunhofer Institute for Applied Information Technology FIT, Schloss Birlinghoven, 53754 Sankt Augustin, Germany

**Keywords:** Reproducibility crisis, Traceability, Trustworthy AI, Federated learning, Personal Health Train

## Abstract

**Background:**

Reproducibility of computational algorithms is a challenging but crucial requirement for medical research and an important component of trustworthy training and application of AI algorithms. Federated Learning (FL) is commonly used to enable privacy-preserving AI in medical research. One prerequisite of reproducibility is traceability. A majority of publications on traceable FL platforms leverage blockchain technology to achieve traceable FL. In healthcare settings, resource-efficient alternatives to blockchains are possible; however, their traceability features require separate design considerations.

**Methods:**

To meet the growing demand for reproducible AI, as outlined in guidelines published by governing bodies such as the European Commission, we propose a novel concept TrainTracks. TrainTracks extends the established Personal Health Train (PHT) Platform for Analytics and Distributed Machine Learning for Enterprises (PADME) to support reproducible and traceable federated learning in medical research. PADME already partially supports tracing the FL and changes to the analysis algorithms used in the FL projects. We extend PADME by adding privacy-preserving change tracing of the data, metadata, and computational experiment execution through integrating it with the tools specialized in distributed data management (DataLad and MetaLad). Finally, we evaluate the proposed concept against the detailed list of requirements to analyze the advantages of the TrainTracks and the need for further design improvements.

**Results:**

Evaluation of the TrainTracks concepts’ compliance with a checklist for reproducible AI showed that TrainTracks improves the original PADME platform in 15 points out of 47 points applicable to FL. The greatest improvement was in data reproducibility, improving 10 of 12 points from no support to full support for automatic information extraction. No improvements were made to method reproducibility, aside from introducing a dedicated reproducibility repository. Experiment reproducibility saw upgrades in 5 of 30 applicable points, mainly through workflow and code traceability.

**Conclusions:**

Our concept of combining FL technology with a data versioning tool provides a structured, automated workflow that traces the FL algorithm itself, delivered algorithms, and the used data. TrainTracks demonstrated high compliance with recommendations for reproducible AI experiments, methods, and data. Our work emphasizes the importance of complete FL process traceability, as all considered aspects individually contribute to the reproducibility of the medical research. Tracking dataset versions in FL is crucial for dynamic application areas, such as medical research, where new data, e.g. electronic health records, are continuously recorded and added.

**Supplementary Information:**

The online version contains supplementary material available at 10.1186/s12911-026-03553-7.

## Background

### Introduction

Medical data is subject to strict privacy regulations, such as the General Data Protection Regulation (GDPR) in the European Union (EU) or the Health Insurance Portability and Accountability Act (HIPAA) in the United States. These regulations require sensitive health data to be handled in a privacy-preserving way. The quality and performance of Machine Learning (ML) algorithms depend on training on large, diverse datasets, which are often distributed across different institutions and are challenging to access due to privacy regulations. The established Personal Health Train (PHT) paradigm addresses this challenge by sending algorithms to distributed data, rather than transferring sensitive data outside of respective medical institutions. Federated Learning (FL) through the PHT enables training and evaluation of ML models across distributed datasets, without the need to transfer sensitive data across institutions, and instead by sharing non-sensitive aggregated results. Our team is involved in the development of the Platform for Analytics and Distributed Machine Learning for Enterprises (PADME) [1], a mature PHT implementation, which has been successfully applied in multiple medical research projects [2–4].

In 2016, M. Baker popularized the term “reproducibility crisis”. After conducting an online survey, 70% of 1576 researchers reported failing to replicate another scientist’s experiment [5]. A lack of reproducibility is also evident in medical research, as more than 60% of surveyed medical researchers reported failing to reproduce others’ results [5]. Especially in a high-risk field such as medicine, this is unacceptable. Reproducible computational research under typical medical research conditions, with continuous alterations and additions to the data calls for appropriate automated documentation on both the data and algorithm sides. R. Peng and S. Hicks [6] analyzed reproducibility problems in computational research in the medical domain, highlighting the need to share data and code in their context and to develop infrastructure to enable reproducible research. Evolving datasets, such as hospital electronic health records, and studies with rolling enrollment are common. As evidenced by over 300 trials identified in a recent review [7], adaptive designs are increasingly used to accommodate accumulating data from evolving datasets, such as electronic health records and rolling enrollment studies.

FL settings are often used to train artificial intelligence (AI) models, making it worthwhile to consider guidelines for AI development support trustworthy computational medical research. Prominent guidelines on trustworthy AI are developed by international consortia to improve the traceability and reproducibility of AI experiments across publication, development, and deployment stages. The Fairness, Universality, Traceability, Usability, Robustness, and Explainability (FUTURE)-AI act [8], published in 2025, focuses on providing technical documentation of the AI models’ training and testing data for improved traceability. The European Commission has published ethics guidelines for trustworthy AI [EU-Trustworthy AI], demanding traceability and reproducibility within their assessment list since 2019. The European Commission demands *traceability* in the way that the datasets, including the data gathering and data labeling, as well as the processes and algorithms used that yield an AI system’s decision, are documented “in the best possible manner” [EU-Trustworthy AI]. Their definition of reproducibility states that AI experiments repeated under the same conditions must exhibit the same behaviour to be considered reproducible. To assess reproducibility, one clearly needs traceable AI experiments in terms of data, methods, and experiments. Specifically in medicine, various research organizations and publishers work towards the AI-specific reproducibility improvements regarding publications by creating checklists, e.g, the Checklist for Artificial Intelligence in Medical Imaging (CLAIM) [9], TRIPOD+AI [10], STARD-AI [11], or CONSORT+AI [12], which specify the quality and completeness of description of the data and code.

Since FL enables the delivery of AI algorithms to sensitive data in medical research, for complete reproducibility, it must be traceable. There has been one recent work by Tariq et al. [13] on gathering information and identifying open issues in trustworthy FL, focusing primarily on traceability but not on reproducibility. However, extensive checklists and guidelines for reproducible medical research using FL are missing. A strong trend toward achieving reproducible AI appears to be the adoption of blockchain technology. As demonstrated by 77.6% of 125 articles on traceable FL in a recent review, mention blockchain technology as the way they ensure traceability [14]. In settings where a central server is trusted to manage updates and aggregation, or where a few trusted nodes are used for which the PHT has been developed specifically, blockchain would add complexity, latency, and storage overhead; more optimal solutions to achieve traceability are applicable.

Albertoni et al. [15] provided a comprehensive summary of recommendations for the reproducibility of AI experiments. The authors broke reproducibility into three categories: experiments, methods, and data, as all are critical. We will build on this work and propose an FL concept that addresses all aspects. The PADME platform already traces methods and aspects of experiments, such as AI algorithms delivered and the FL-specific algorithm. To ensure data reproducibility, we propose extending the PADME platform by integrating it with data versioning and metadata via the distributed data management system DataLad. Finally, we will evaluate how our extension conforms to the work of Albertoni et al.

### Methodical background

In this section, we will introduce the technical details of the tools we will combine in the concept to enable reproducible medical research using FL. Subsection 1.2.1 will introduce the Personal Health Train (PHT) and the PADME platform, the FL concept, and the FL platform we use. Subsection 1.2.2 introduced DataLad and MetaLad, tools that we use to add trace changes to the datasets used in an FL project.

#### PHT and PADME

The Personal Health Train (PHT) is a distributed analytics concept, developed specifically for the medical domain. The PHT was introduced in 2020 [16] with a history of successful implementations [2–4]. The PHT concept consists of three components: Stations, Trains, and the Central Service [16]. The Stations represent the different data nodes visited by the Train. The Trains transport analytical tasks, via the network, to each Station. The Central Service coordinates Train schedules and aggregates distributed analytics results. PADME is an implementation of the PHT concept.

In PADME, the data analysis code is encapsulated within a Docker image, enabling self-contained execution of the code transported by a Train. Trains are pulled by the Central Service from Harbor (harbor.io), a repository for Docker images, that serves as a Central Train Repository [1, 17]. The network used by the Trains is an architectural concept; whether that means the internet, a virtual network, or a local network depends on the specific deployment of PADME. To improve the compliance with Findable Accessible Interoperable and Reusable (FAIR) principles [18], PADME uses the Distributed Analytics Metadata Schema (DAMS) to monitor information on Stations (e.g., the owner, references to the domain-specific metadata) and Trains (Train image identifier, central unit processing consumption, the expected data format) [1, 19]. This information is saved within each Station and in the “metadata store” within the Central Service.

FL projects carried out via the PADME platform follow a typical workflow, in which a researcher’s plan and analysis are approved by a committee and by each Stations admin. During this process, the Station Admins can approve or deny operations on their local data as well as all data that leaves their corresponding Station. Any researcher wanting to conduct an FL project must submit a research proposal, including the intended data analysis algorithm and the Stations they apply to, for approval by the committee.

#### DataLad and MetaLad

DataLad is a well-documented Python-based distributed data management system. It is a tool that is established and commonly used in the neuroscientific community, as it is used by the Canadian Neuroscience Platform [20], the Brain Research Through Advancing Innovative Neurotechnologies (BRAIN) initiative archive [21] and Nationale Forschungsdaten Infrastruktur Neuroscience (NFDI-Neuro) initiative [22]. DataLad provides version control for distributed data, code, and their relationships. The version control is based on Git, a popular distributed version control system for software development, and Git-Annex, a tool that tracks file versions via references [23]. As it is a Git-based tool, DataLads can be nested, consisting of super and sub DataLads. A dataset version can be created with Datalad save version-tag “version-number”, which is similar to a git commit: it creates a snapshot of the data and associates a version number with it. Apart from manually creating save messages, DataLad also allows automatic linking to scripts that alter the contents of DataLad.

The DataLad Handbook recommends following the Yale University Open Data Access (YODA) principles. Whenever a dataset is useful in more than one context, YODA recommends treating it as its own entity. Treating a dataset as its own entity means one should also track where it came from, how it was processed, and where it is now. According to the YODA principles, the DataLad team proposes a folder and dataset structure comprising input, output, code, and environment folders [24].

MetaLad is an extension of DataLad [23] that allows associating metadata with datasets and files, extracting metadata from primary data to separate metadata files, and searching for and transporting associated metadata. Metadata extraction is performed using MetaLad extractors, scripts that generate JSONs conforming to MetaLad’s schema. Some extractors are provided by MetaLad, but users can write their own and register them with MetaLad. The extracted metadata can be stored in a git repository and must include whether it is associated with a file or a dataset, an identifier, the dataset version, the metadata extractor used, the agent supplying the metadata, and, of course, the metadata itself. This metadata can then be added to a DataLad dataset via a specific command [25].

## TrainTracks – the concept

**Fig. 1:**
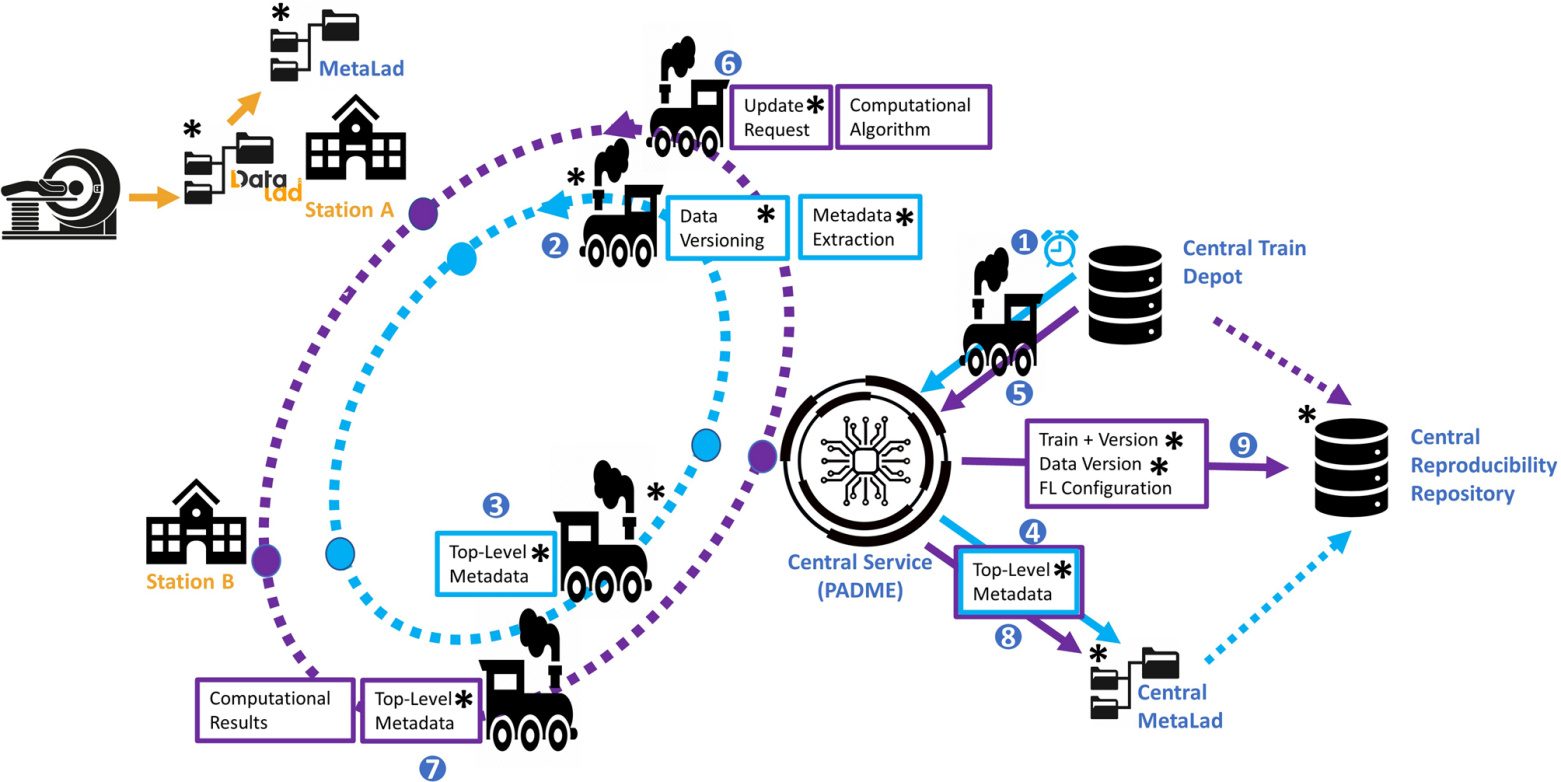
Visual representation of TrainTracks. Here, the blue arrows represent a regularly scheduled data Versioning Train, whereas the purple arrows represent the dataflow in any Analysis Train, adjusted to TrainTracks. The numbers indicate the order in which the actions are performed. A star (*) indicates components that are not part of the original PHT paradigm

TrainTracks is a concept extending PADME by setting up local data versioning infrastructure at each Station, a Data Versioning Train, and a Reproducibility Repository at the Central Service. An overview of the processes is shown in Fig. 1. Each Station sets up a DataLad dataset for its data, following the YODA directory structure. The Station-specific datasets are accompanied by local git repositories that store metadata extracted with MetaLad. Each Station’s local DataLad and MetaLad are hosted locally within the Station’s secure network. TrainTracks strongly relies on a so-called Data Versioning Train (Fig. 1), which executes a DataLad save command, extracts the metadata, and commits it. The Data Versioning Train transports metadata describing the aggregated dataset of all involved Stations to the Central Service (Fig. 1), where it is saved in the Central MetaLad (Fig. 1), while referencing the dataset version. This aggregated metadata is cleaned of sensitive information. The Central MetaLad is a repository hosted by the Central Service that stores versioned, non-sensitive metadata for the data at the local Stations. The local Stations MetaLad repositories are not connected to the Central MetaLad repository. The metadata from the Central MetaLad can be regularly streamed to an external public data catalog to improve the findability of the data used during the FL project. Any regular Analysis Train will be extended, with a Dataset Versioning Script, with the same functionality as the Data Versioning Train (Fig. 1–1). After an Analysis Train run, the version of each dataset the Analysis Train was run on, the Analysis Train version and the Analysis Train configuration will be stored in the Reproducibility Repository, a relational database, within the Central Service (Fig. 1).

Extending the Data Analysis Trains this way enables reproducibility of the analysis carried out by the Data Analysis Train. A separate, regularly scheduled Data Versioning Train keeps the Central MetaLad up to date, whereas Data Analysis Trains are only run when a researcher requests them. A regularly updated Central MetaLad makes the aggregated dataset available to the PADME project consortium and makes it findable. Additionally, a regular Data Versioning Train can detect changes to the dataset, enabling workflows to rerun Data Analysis Trains on the most recent dataset version, thereby enhancing support for dynamic data analysis in the medical domain.

TrainTracks has to be enabled by extensive harmonization and standardization across the different Stations. The harmonization should be supported by unified workflows and configuration templates for the file structure, but needs to be accompanied by manual coordination throughout data addition. We provide more details on the dataset structure requirements and metadata-related issues in Subsects. 2.1 and 2.3. At this point, we want to highlight that the harmonization across the Stations in our TrainTracks is mostly a manual coordination process that should be supported by designated data stewards, data management plans, and standard operating procedures (SOPs).

Currently, the PADME project committee already approves and rejects project proposals. If a researcher submits an AI project to them, they should extend their check to include reproducibility aspects of the study design and the suggested algorithm.

### Example

To illustrate TrainTracks, each of the following sections will be accompanied by the following example: a joined PADME project between the department for neurology of Cologne and of Frankfurt, with the goal to develop an AI algorithm to localize and identify the type of a stroke (ischemic vs hemorrhagic) via Magnetic Resonance Imaging (MRI). We will present an exemplary setup for the local Station in Cologne, where newly recorded MRIs that meet the project’s inclusion criteria will be immediately added to the local Station’s dataset. We will also train a convolutional neural network (CNN) for that purpose in the FL setting.

### Structure of the dataset

**Fig. 2:**
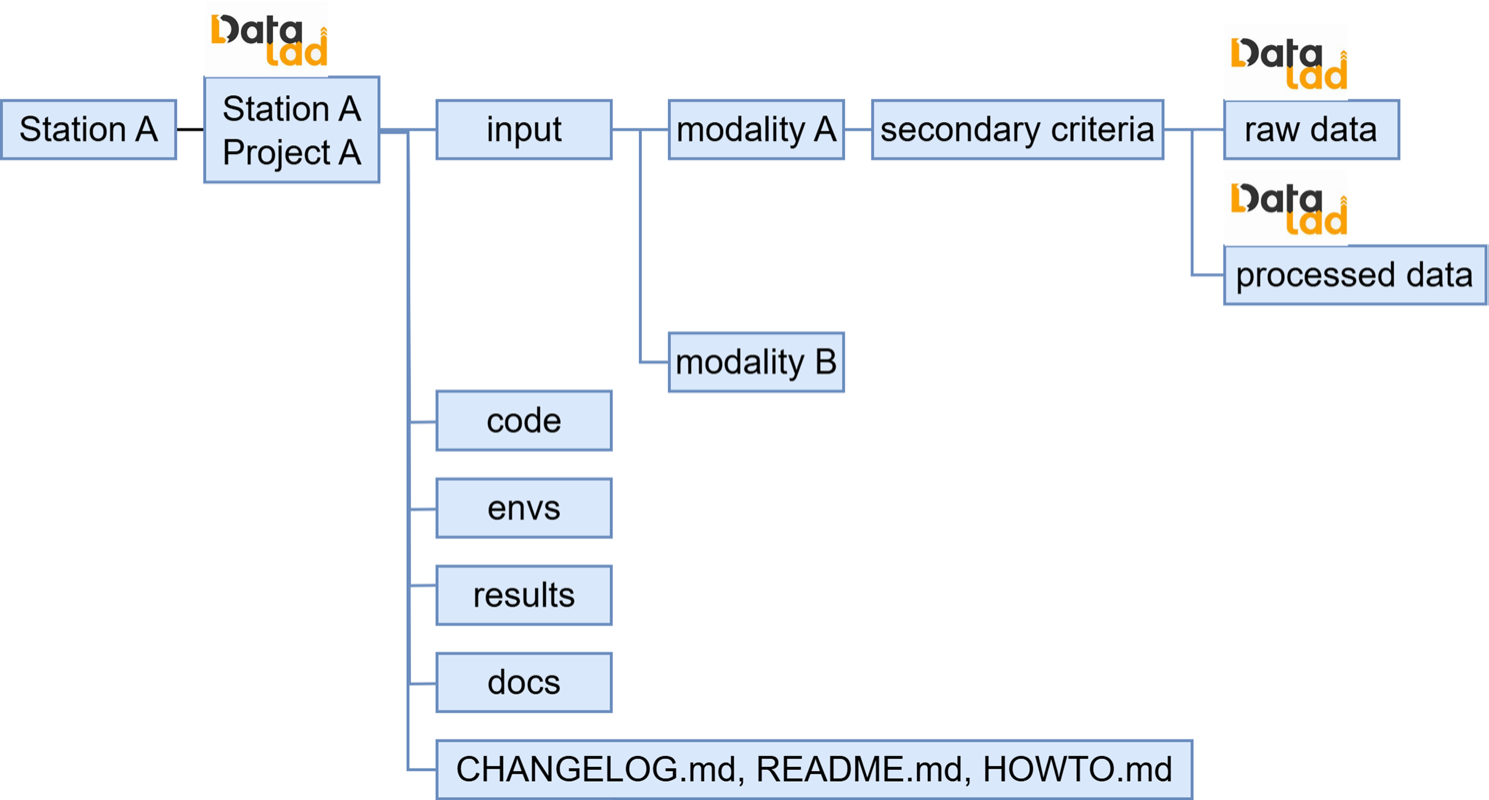
The local and central DataLad structure of the datasets. For each Station, the data for the FL project are organized into a single project-specific DataLad dataset, labeled Station a project a in the figure. This local top-level dataset follows the YODA principles of separating input, output data, and processing steps. The dataset’s subsets are divided by modality, potentially further divided by a secondary criterion. Finally, they are further subdivided into raw and processed data. The division of these sub-datasets may be project-specific, but it should be ensured that for all involved Stations it follows the same logic. Finally, the bottom-level folders containing the actual data are DataLad datasets, and DataLad sub-datasets of the Station a project a DataLad dataset

Inspired by the specific implementation of the YODA principles [24] by Kalantari et al. [26], we propose a data file structure for Stations to follow (Fig. 2). Apart from the input data, the DataLad instance also contains pre-processed data, local outputs, local scripts, and additional documentation. Then datasets should be grouped in layers. First, they should be grouped by modality, in the sense of data type and acquisition protocol, e.g., images, reports on a subject’s behavior, and histology reports. Secondly, the data should be grouped, e.g., by experiment setup or subject groups, depending on the community-specific needs. At the lowest layer, the data should be grouped by their pre-processing level. Each low-level pre-processing folder should be tracked as a single DataLad subdataset. Additionally, the folder that groups all input data, code, environments, results, and documentation for the FL project should be the super DataLad dataset. This is the Station and project-specific dataset. The general structure of all the Station and project-specific datasets should be the same and is agreed on by all project partners. To support standardization and harmonization across stations, the PADME project committee should provide this folder structure as a configuration template.

#### Structure of the dataset - example

When MRI images are recorded, they are typically stored in Digital Imaging and Communications in Medicine (DICOM) format in a Picture Archiving and Communication System (PACS). In the neurology department in Cologne, images are stored in the open-source PACS Orthanc. The department has developed a workflow and scripts to export datasets from that Orthanc to a DataLad dataset [27]. This workflow results in a DataLad repository that manages DICOM and exposes selected DICOM header fields to enable metadata sorting and filtering. In the example, the committee agrees to store the MRI data, sorted primarily by protocol name and secondarily by the MRI series number. As protocol names may differ between Cologne and Frankfurt, the committee publishes a mapping that project members must follow. An automatic mapping script that renames the protocol name has been implemented and applied in Cologne. Here, each dataset created by dividing by protocol name should be treated as a sub-dataset, as it can be useful across different research projects. Once the DataLad repository is initialized, the Station admin tags it as version 1 (“v1.0”), and subsequent transformations will be recorded via DataLad using incremental versioning. The committee takes inspiration from the Brain Imaging Dataset Structure (BIDS) [28] and asks the local Station admins to create a BIDS-compliant dataset in the subdataset “raw”.

**Fig. 3:**
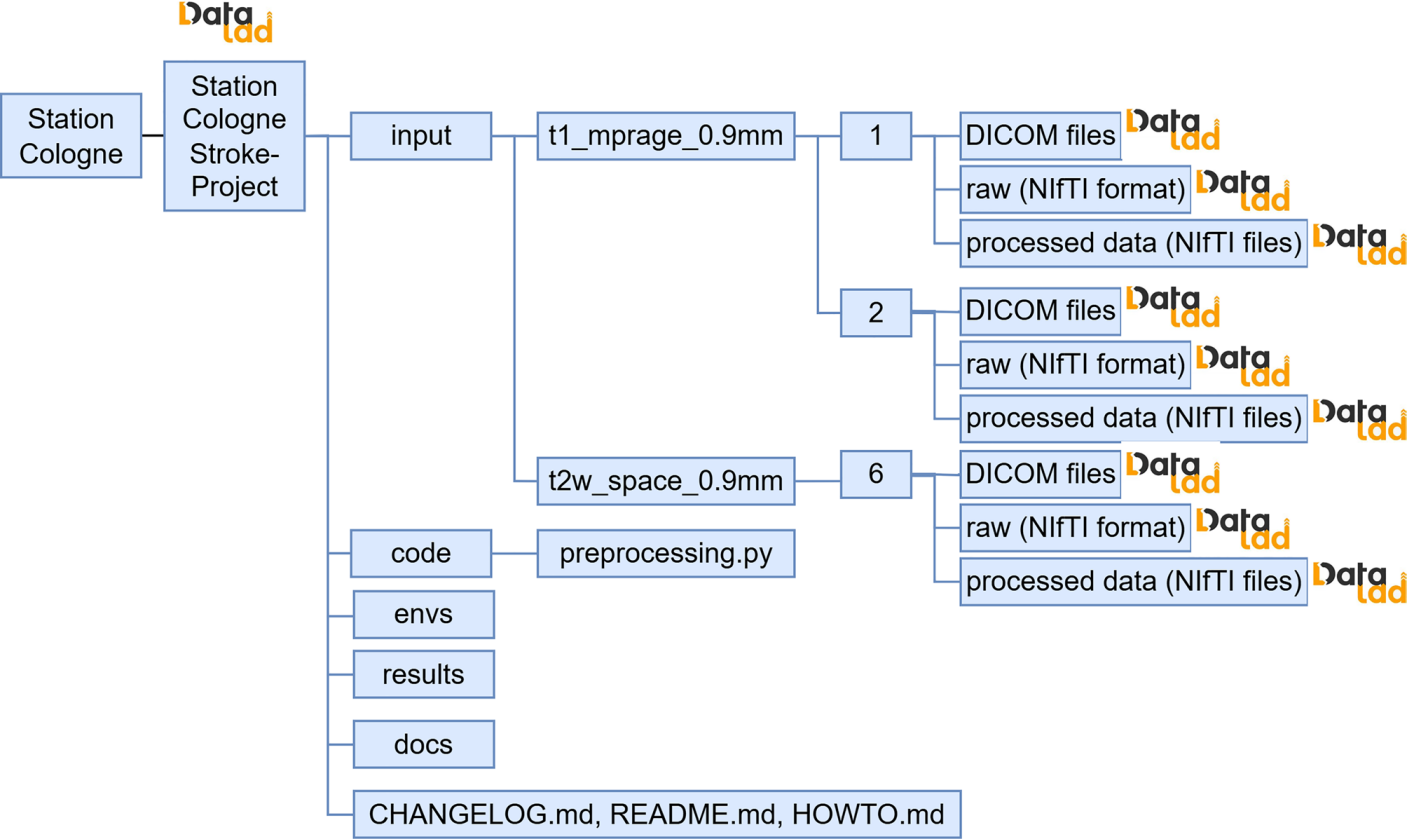
Example instantiation of the DataLad structure

To avoid incompatibilities of the MRIs themselves, Cologne and Frankfurt apply suitable preprocessing steps. These site-specific preprocessing steps are run and documented via DataLad. Data sorting and application of pre-processing steps result in the file structure shown in Fig. 3. Each of the “DICOM files”, “raw”, and “processed” folders is a separate DataLad dataset that is included in the Stations project-specific super-DataLad dataset.

### Metadata repository and extractors

**Fig. 4:**
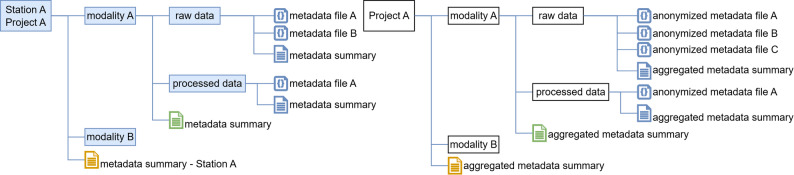
The structure of the metadata repositories at the local Stations (in blue) mirrors the structure of the DataLad datasets, but leaves out folders that do not contain datasets. Each box represents a repository. The metadata repository of the FL project in the Central Station contains anonymized metadata on the file level and aggregated metadata summaries on the dataset level

The metadata tracked by MetaLad, different from DataLad datasets that come with repository support, needs to be stored in a dedicated git repository. Our proposed structure is shown in Fig. 4. The Station-specific metadata repositories mirror the structure of the folder within the DataLad input folder. Since the sub-datasets have been created according to the YODA principles, they are standalone datasets that may be useful for other projects. So, each of these standalone datasets should have its own metadata collection in its own repository, enabling its findability and reuse. To support highly community- and context-specific, data-type-specific descriptions of datasets, each project can freely choose the metadata format that best fits its needs. However, to comply with the FAIR principles, the same metadata format should be used for the same type of data throughout the project. The metadata for each standalone dataset should include metadata describing the individual files it contains, as well as metadata describing the dataset in its entirety. To avoid information duplication, the Station- and project-specific super repositories should contain only metadata files that describe their structure, provide context to understand the dataset, and include additional information, such as study setup, that is not already captured in their sub-repositories. Based on these individual files and dataset metadata, the Data Versioning Train can generate a machine-readable summary describing the data at each station. This summary should include the contact person for the dataset. After cleaning, this summary of sensitive information will be compiled into a single metadata file describing the entire input dataset for the Station. The file-level metadata will be anonymized. This summary metadata will be transported, alongside the individual anonymized file-level metadata, to the Central Service by the Data Versioning Train and attached to the Central MetaLad. When the Train leaves the local Station, the Station admin needs to approve the metadata summary as a final checkpoint. From the Central MetaLad, metadata can be optionally published in the project committee’s chosen data catalog. To ensure data reproducibility by tracking the Stations dataset versions, the aggregated metadata includes a dictionary of recent DataLad save-identifiers.

To enable metadata tracing and publishing, a suitable MetaLad extractor for the project’s files and file structure must exist. These extractors may be obtained from the MetaLad extractor library or written specifically for the project. As PADME projects are generally carried out within a committee, it is straightforward for the committee to select suitable metadata standards for the different metadata levels within their projects. If needed, the committee would also be responsible for developing the corresponding metadata extractors. The metadata schema capturing the aggregated top-level metadata of all participating Stations should be chosen keeping compliance with data protection laws, like the GDPR and HIPAA, in mind. It is advisable to choose metadata standards that include existing, proven anonymization guidelines and profiles.

#### Metadata repository and extractors – example

**Fig. 5:**
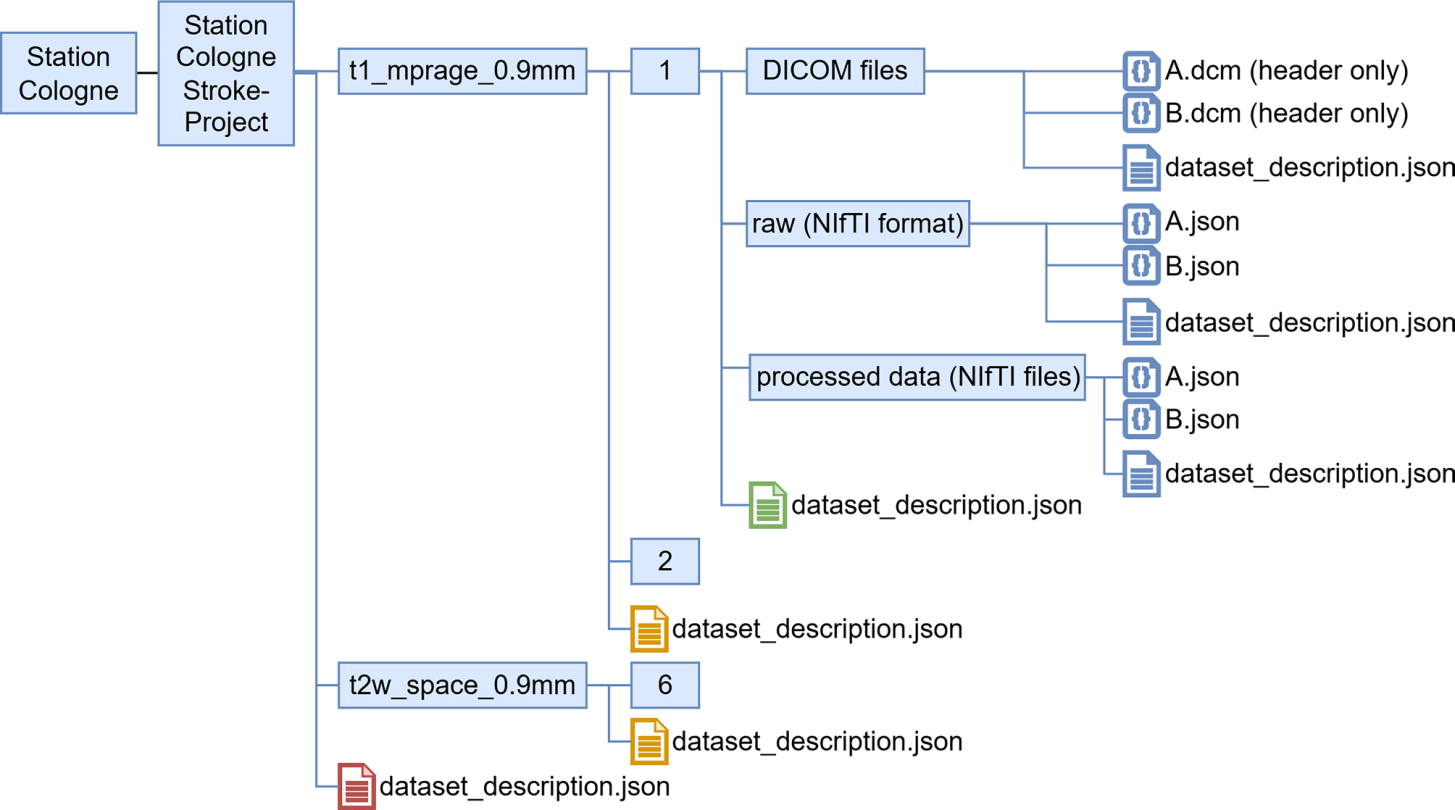
Exemplary structure of a local MetaLad repository structure

Cologne and Frankfurt teams both collect MRI in DICOM format and save it in the same file structure, as explained in Sect. 2.2.1. Despite differences in specific DICOM implementations between Cologne and Frankfurt, only DICOM header fields relevant to the project require standardization between both stations for aligned metadata extraction, simplifying dataset integration. Since each DICOM file already contains its own metadata, no additional metadata describing each file is needed; however, depending on project requirements, further metadata can be added flexibly. Same for the data files saved in the “raw” sub-datasets within BIDS-compliant format, since each file is accompanied by a sidecar JSON file. Dataset-level information can then be exported through DataLad in the BIDS schema. Developed metadata extractors to automatically create dataset-level information from a set of DICOM files and from a set of BIDS conforming sidecar files. The metadata files and structure tracked by the local Station MetaLad repository are shown in Fig. 5.

### The data versioning train

The Central Station is the actor that coordinates and schedules the trains within the PHT framework, PADME. As such, it triggers the Data Versioning Train. In TrainTracks, the Data Versioning Train is visiting all Stations of the FL project according to a pre-set, project-specific regular schedule. Thus, regularly informing the Central Service, which may react to the detected dataset changes. Apart from access to the local dataset, the Data Versioning Train has access to the local DataLad. Before the Data Versioning Train is run for the first time, the Central MetaLad is instantiated with a metadata file describing an empty dataset. This “aggregated metadata file” will be filled by the Data Versioning Train and describe the aggregated dataset of the Stations.

**Fig. 6:**
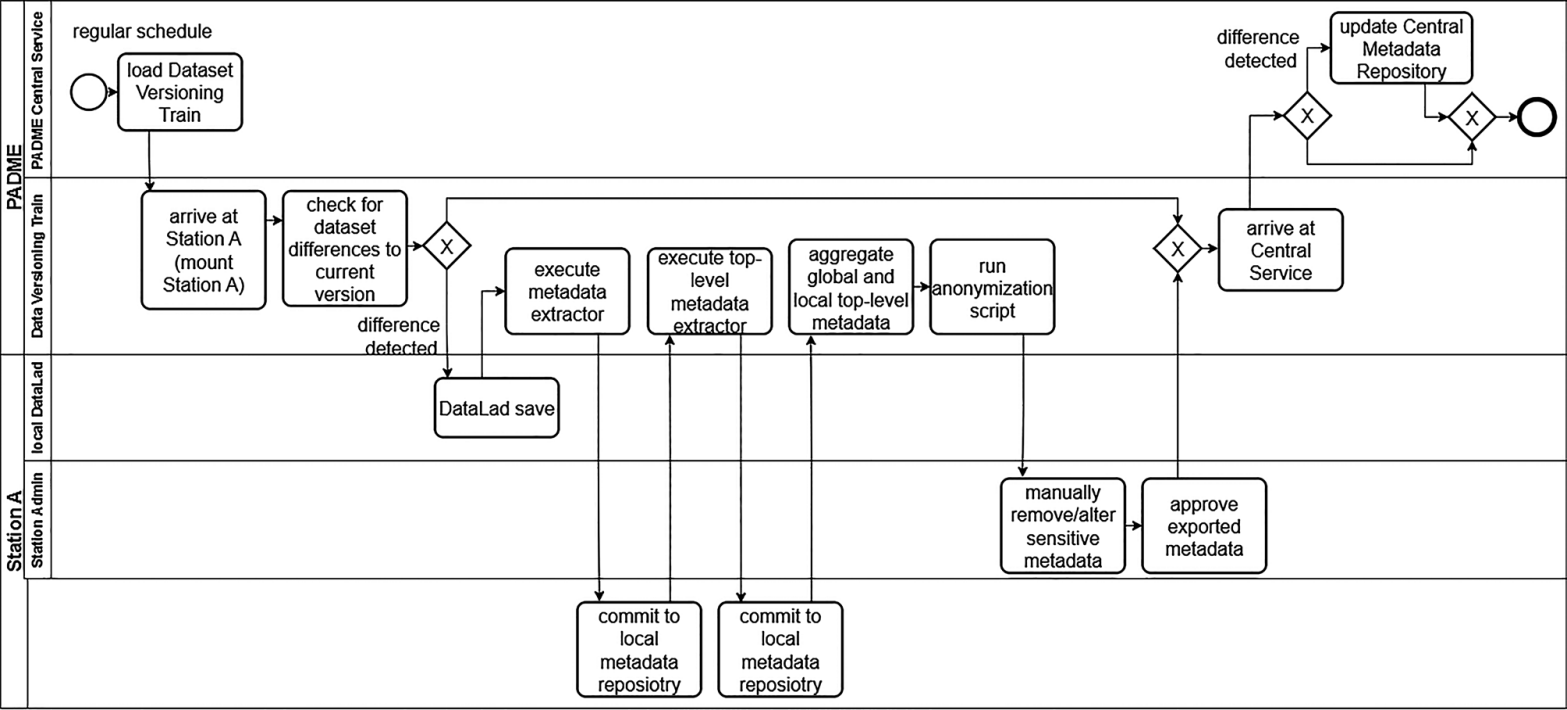
Activity diagram showing the workflow of a scheduled data Versioning Train for a single Station A

The detailed workflow of the Data Versioning Train is shown as an activity diagram in Fig. 6. Once scheduled, the Data Versioning Train visits each Station in the order specified in the train’s configuration. The Central Station loads and dispatches the Data Versioning Train. At each Station, the local DataLad is mounted to the Data Versioning Train. The Data Versioning Train compares whether there are differences between the datasets’ last saved versions in the local DataLad and the current dataset. If differences are detected, the dataset is versioned by a DataLad save initiated by the Train. Additionally, the metadata extractor for local metadata is executed on the Stations data. This way, metadata on a fine-grained level, which may contain sensitive information, is generated, outdated metadata files will be replaced, and finally, the up-to-date metadata is committed to the local MetaLad repository. A second metadata extractor will generate one top-level metadata file describing the dataset at the current Station in its entirety. The resulting top-level metadata is also committed to the local MetaLad repository. Based on this top-level metadata file, the Data Versioning Train will update the “aggregated metadata” with the information about the Stations dataset. Next, the Data Versioning Train will run an anonymization script on the aggregated metadata in order to automatically remove sensitive information. This anonymization script should be developed and maintained by the PADME project’s committee. As a final safety measure, the Stations admin inspects the aggregated metadata, the admin may manually alter the aggregated metadata without falsifying information, and gives final approval. Finally, the updated dataset version and aggregated metadata will be transported by the Data Versioning Train to the Central Service.

We chose to use the Train to transport this information instead of linking the Stations MetaLads under one Central MetaLad, as it is a network connection that is already established securely and approved by stakeholders, e.g., the information technology department of the hospital. After the Train has visited all scheduled Stations and returned to the Central Service, the Central Service checks whether any changes were made to the datasets. If the datasets changed, the central MetaLad repository is updated.

As mentioned in Sect. 2, the metadata of the central MetaLad repository may be published, improving the findability of the project’s aggregated dataset. Via these regularly scheduled dataset updates, the Central Service can detect changes in the dataset and trigger a rerun of FL on the updated data, ensuring the timeliness of the data analysis.

#### The data versioning train – example

The PADME project committee decides to schedule the Data Versioning Train once a week. Because MRIs are continuously recorded in Cologne and Frankfurt, the committee does not want to miss when a new recording of a stroke patient is added to the dataset. The Data Versioning Train uses the metadata extractor supplied by the committee, as introduced in Sect. 2.3.1. Anonymization scripts are executed on the DICOM headers, sidecar files, and aggregated dataset-level metadata files before requesting approval from the Station Admin. Once the Data Versioning Train reaches the Central Station, it commits the metadata files to the Central Metadata Repository.

### Tracing data analysis trains

**Fig. 7:**
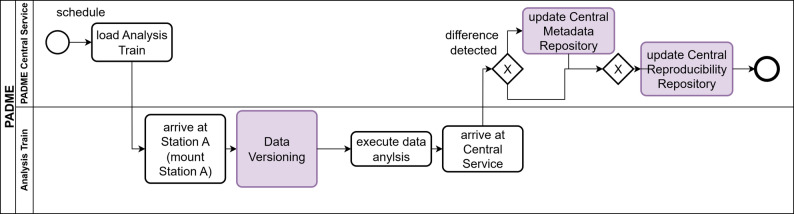
Activity diagram of the extended Analysis Train. The parts TrainTracks added to a typical data Analysis Train are marked in purple

After ensuring that changes to datasets at the Stations can be traced and their metadata documented by setting up appropriate structures at the Stations, the next challenge is making the FL algorithm itself traceable. For this purpose, the Analysis Train is extended as shown in Fig. 7. Each Data Analysis Train requests the current dataset version at each Station it encounters. These will then be saved as Station-Dataset Version identifier pairs within the Data Analysis Train and transported back to the Central Service. Before a Data Analysis Train runs the computational algorithm, it will run a Data Versioning Script to check whether any changes have occurred since the previous dataset versioning. If needed, a new dataset version is created this way. If a Data Analysis Train produces output at any Station, this will be followed by a DataLad save that references the producing Data Analysis Train and its version.

#### Tracing data analysis trains – example

A researcher from Frankfurt regularly checks the Central Metadata Repository to determine whether the PADME project has collected enough MRIs that meet their studies’ inclusion criteria. Once enough MRIs have been collected across the two sites, the researcher applies to the PADME project committee to train a convolutional neural network to localize a stroke. After the committee approves the machine learning project, the researcher configures the FL framework to meet their specific needs. They containerize their code and configure an FL project that trains one epoch per Analysis Train run. For 50 epochs, there are no updates to the dataset; the Analysis Train runs as usual, with one addition: after each run, the Central Reproducibility Repository is updated with information concerning the previous run. At epoch 51, the Data Analysis Train detects that a new MRI has been added to Cologne’s local Station. It then executes Data Versioning and data analysis as usual, training only on the original data it has been configured to run on. Once the Data Analysis Train reaches the Central Station, the researcher will be notified of the dataset change. They decide to incorporate the new data from the beginning into the FL project, so they restart it.

### Reproducibility repository

At the Central Service, a trace of the Data Analysis Train run will be created and saved in the Central Reproducibility Repository. The Central Reproducibility Repository can be realized as a relational database. This trace contains:

- a reference to the Data Analysis Train on the Central Train Repository and the version tag
- the complete list of parameters from Data Analysis Train configuration, e.g., whether it is run in FL or incremental learning mode, the aggregation strategy and the Data Analysis Train schedule
- a dictionary containing the dataset version number of the analyzed dataset at each Station

As DAMS [19], a metadata schema for the FAIRification of distributed analysis within the PHT concept has already been developed; it is a natural candidate for capturing the aforementioned concepts as well. Each of these reproducibility traces should be stored within the PADME Central Service for future reference.

#### Reproducibility repository – example

The researcher trained a high-performing stroke localization algorithm using TrainTracks and now wants to publish their results. Using the Central Reproducibility Repository of TrainTracks, they can describe the FL project in great detail and generate artefacts that improve the reproducibility of their paper.

## Results

The proposed concept (TrainTracks) is evaluated against the criteria listed in the manuscript by Albertoni et al. [15], which summarizes various guidelines for reproducible research with AI methods. The authors provide checklists for reproducibility of data, methods and the experiment itself. In addition, the work provides recommendations for reproducible AI in biomedical research. It is important to note that [15] specifically focuses on reproducibility as detailed reporting when publishing. Our work shifts the focus to the previous step, namely, on how well the reproducible publication process can be supported by automatically extracting information. First, the points not applicable to FL were excluded. Then, we assessed the original PADME platform’s compliance with the listed reproducibility requirements and analyzed the improvements provided by the TrainTracks concept.

We evaluate our concept (TrainTracks) on the three checklists of [15] on data, method, and experiment reproducibility, and briefly discuss the recommendations specific to deep learning and biomedical applications. Each category is accompanied by a table containing a point-by-point evaluation. The complete tables, including reasoning for each point, are provided in the digital supplementary file “Additional File 1”. Each point is evaluated as *not applicable*, *manual support only*, *partial support,* and *full support*. Some points are not applicable to the FL setting, e.g., due to data sensitivity. If no automatic support (it can be fulfilled without manual intervention by the researcher, Station admin, or committee) is provided for any subpoint of a point, it is evaluated as manual support only. A point is evaluated as partial support if not all subpoints of a point are supported in an automatic manner, whereas a point is evaluated as full support if each subpoint is supported in an automatic manner within the platform.

### Data reproducibility

The manuscript [15] lists 17 points that need to be fulfilled to achieve full data reproducibility. Table 1 shows those points and the evaluation of PADME and TrainTracks against them. Of the points, nine are fully applicable, and three are partially applicable to FL. PADME itself does not support any of these 12 points. TrainTracks improved 10 of those 12 points on data reproducibility due to its properties, such as enforcement/support of the YODA principles, augmentation of data with metadata, and automatic versioning of datasets via Data Versioning Trains and extended Analysis Trains. Additionally, TrainTracks already puts in place infrastructure for tracing changes to data made via a script, encouraging its use.

**Table 1:**
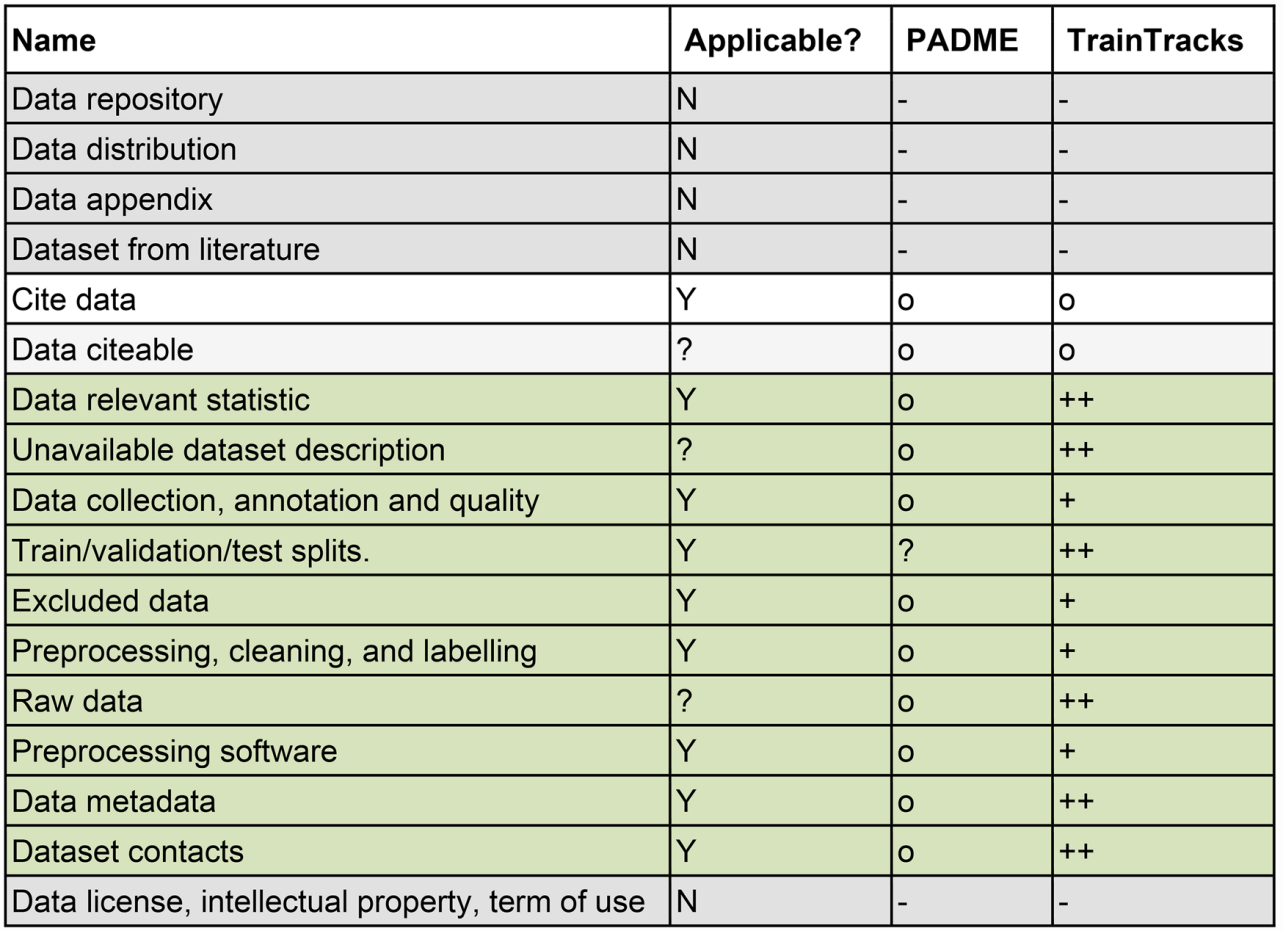
Evaluation of support for data reproducibility according to [15]

**Legend**: (-: not applicable; o: manual support only; +: partial support; ++: full support) (grey background marks points not applicable to FL absolutely, green rows mark points where TrainTracks improved the support provided to PADME). Applicability in the FL setting is indicated by “Y” (applicable), “N” (not applicable), or “?” (applicability depends on the specific case)

### Method reproducibility

To ensure method reproducibility [15], provides 10 points, all of which are fully applicable to the FL setting. Table 2 shows these points and the evaluation of PADME and TrainTracks against them. The checklist for method reproducibility contains four points on describing the research question, hypotheses, problems, and study design. These four points are not supported by PADME itself, as FL is simply a chosen tool to support the study. Since the models and algorithms are available within the Analysis Trains run, all run configurations, including aggregation strategy and schedule, are already saved within PADME Central Service; points regarding the models and algorithms are partially supported. PADME partially supports the ten points for full method reproducibility as is. In TrainTracks, the run configurations are additionally stored in the Central Reproducibility Repository. However, this additional layer does not upgrade the support for these six points to full support. Full support can be achieved by adding a software analysis tool, such as Pseudogen [29] or Codemeta Harvester [30].

**Table 2:** Evaluation of support for method reproducibility according to [15]

Name	Applicable?	PADME	TrainTracks
Hypothesis	Y	o	o
Prediction	Y	o	o
Setup	Y	o	o
Problem description	Y	o	o
Outline	Y	o	o
Model involved	Y	+	+
Method assumptions and restrictions	Y	o	o
Mathematical setting	Y	+	+
Algorithm complexity	Y	+	+
Pseudo code	Y	+	+

Legend: (-: not applicable; o: manual support only; +: partial support; ++: full support) (grey background marks points not applicable to FL absolutely, green rows mark points where TrainTracks improved the support provided to PADME). Applicability in the FL setting is indicated by “Y” (applicable), “N” (not applicable), or “?” (applicability depends on the specific case)

### Experiment reproducibility

Regarding experiment reproducibility by [15], 27 points (out of a total of thirty) are fully applicable, and three points are partially applicable to FL. Table 3 shows those points and the evaluation of PADME and TrainTracks against them. No support is provided by PADME and TrainTracks for points addressing the interpretation of the analysis results (1 point), assumptions (1 point), claims (2 points), citations (1 point), and points addressing designing the experiment that go beyond documenting it (2 points). Another point not supported by PADME and TrainTracks is metadata, publication, and licenses of the code itself (5 points). For five points, TrainTracks upgraded PADME from partial to full support by extending the Train run information to include the exact Train version and the Stations dataset version. Since TrainTracks enforces local dataset structures that comply with the YODA principles and supports tracing of data processing steps via DataLad, workflows for raw, processed, and final data can be traced, thereby improving this from no support to full support.

**Table 3:**
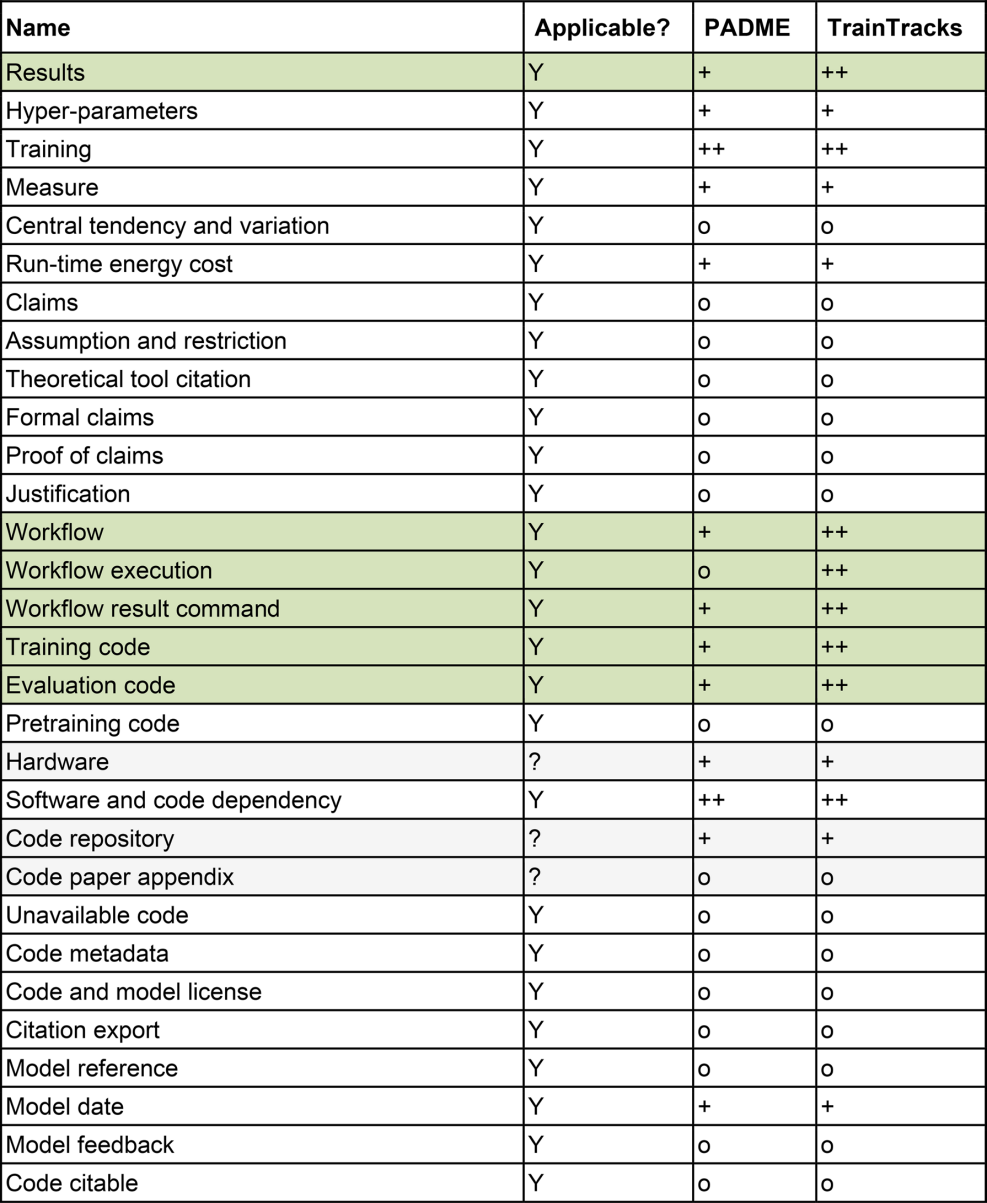
Evaluation of support for experiment reproducibility according to [15]

Legend: (-: not applicable; o: manual support only; +: partial support; ++: full support) (grey background marks points not applicable to FL absolutely, green rows mark points where TrainTracks improved the support provided to PADME). Applicability in the FL setting is indicated by “Y” (applicable), “N” (not applicable), or “?” (applicability depends on the specific case)

### Machine learning and deep learning specific recommendations

Apart from recommending points for data, methods, and experiment reproducibility [15], also presents recommendations specific to deep learning, deep/reinforcement learning in embodied agents, and AI development in biomedical applications. Each proposed point is discussed in detail in Appendix 1.

16 of the 21 proposed points (marked in blue in Appendix 1) address the design of the FL project, including algorithm selection, data curation, and model evaluation. From our point of view, it is not within the scope of an FL platform to evaluate and assess project design, especially in the medical context. Since we do not want to “pick-and-choose” which points we evaluate TrainTracks against, we chose not to evaluate It against any of the ML- and DL-specific recommendations. Each project that uses the TrainTracks infrastructure is initiated by a proposal that is either approved or denied by a committee. We recommend that the committee incorporate the recommendations of [15] in their decision process.

## Discussion

We proposed TrainTracks, a concept for an FL platform, that tracks the three components of reproducible research with AI as listed in [15]: data, method and experiment. We improved the PADME platform in two aspects. Firstly, we added a layer of local dataset versioning and automatic metadata extraction. Secondly, we extended the platform to trace the FL itself within a Reproducibility Repository. TrainTracks added partial or full support to 10 of 12 points to achieve full data reproducibility, via integrating tools already popular within specific medical research communities and providing automatic versioning of data at local Stations. Additionally, we improved the method reproducibility of the original PADME platform by tracing the exact version of the analysis code run at each station. By adding support for dataset versioning and tracing the exact data used by an FL run, the concept would improve experiment reproducibility. Overall, 15 points of the checklist defined in [15] were improved, demonstrating the value TrainTracks can bring to reproducible medical research using FL. There are still gaps in experiment reproducibility, mainly regarding the AI algorithms delivered by FL. One way to improve this is to enforce the use of metadata describing Analysis Trains. Further support for this can be given by integrating a metadata extraction script when uploading an Analysis Train to the Central Train Depot. At the upload stage, a step to check whether the Analysis Train follows reproducibility recommendations, such as seeding random parameters and reporting analysis results’ variance, should be added in future work.

One limitation of TrainTracks is linked to the nature of DataLad, which was initially designed for file-based data sets, commonly used within the computational neuroscience community where the solution originated. Because of this design, there is no inherent way to track the version of data stored in a database management system, e.g., relational or non-relational databases such as PostgreSQL or MongoDB. Building transition layers and exporting the database contents to a file system to enable the solution’s functionality is straightforward, but looking for possible alternatives before proceeding with the implementation is a necessary step for further work.

An evaluation on computational complexity, energy consumption, and needed storage should be provided for a concrete implementation.

## Conclusions

In this work, we propose TrainTracks, a concept that integrates the PHT paradigm and data versioning with DataLad to enable automatic tracing of dataset changes and FL algorithm execution. This way, we addressed the reproducibility of medical research using FL. TrainTracks extends local Stations infrastructure with DataLad and MetaLad, regularly scheduled automatic dataset versioning, and metadata extraction via Data Versioning Trains. TrainTracks enables local Station admins to automatically version their local data using DataLad, a tool already established in the medical research community. We extended the Central Service with a Reproducibility Repository that keeps detailed traces of each run, including information on the dataset and training version, offering a storage-efficient alternative to blockchain-based approaches to improve FL traceability. This way we achieve:

- regular and automatic versioning of distributed data, addressing the challenge of reproducible results on continuously changing datasets typical to medical research
- reproducibility of all three aspects: methods, experiment, and data, by tracing the configuration of the executed FL algorithm, the computational analysis delivered via FL, and the exact data used
- regular and automatic updates to a central metadata repository, enabling all project partners to have an overview of the data available and take informed decisions on the choice of algorithm and the status of the conducted study
- sole reliance on DataLad, MetaLad, and PADME, tools already established and approved by the medical research community

In future work, we aim to implement TrainTracks with researchers from the neuroscience community, who are already familiar with DataLad, for the use case presented in the example.

## Electronic supplementary material

Below is the link to the electronic supplementary material.

Supplementary Material 1

## Data Availability

The datasets analysed during the current study are available in the PADME development GitLab repository at https://git.rwth-aachen.de/groups/padme-development, the DataLad GitHub repository at https://github.com/datalad/datalad, and the MetaLad GitHub repository at https://github.com/datalad/datalad-metalad.

## Abbreviations

FL: Federated Learning
ML: Machine Learning
PHT: Personal Health Train
PADME: Platform for Analytics and Distributed Machine Learning for Enterprises
EHR: Electronic Health Record
GDPR: General Data Protection Regulation
EU: European Union
HIPAA: Health Insurance Portability and Accountability Act
AI: Artificial Intelligence
FUTURE: Fairness, Universality, Traceability, Usability, Robustness and Explainability
FAIR: Findable Accessible Interoperable and Reusable
DAMS: Distributed Analytics Metadata Schema
YODA: Yale University Open Data Access
CNN: Convolutional Neural Network
BIDS: Brain Imaging Dataset Structure
MRI: Magnetic Resonance Imaging
DICOM: Digital Imaging and Communications in Medicine
PACS: Picture Archiving and Communication System
BET: Brain Extraction Tool
MNI: Montreal Neurological Institute
BRAIN: Brain Research Through Advancing Innovative Neurotechnologies
NFDI-Neuro: Nationale Forschungsdaten Infrastruktur Neuroscience
